# Preliminary results of a vaginal constraint for reducing G2 late vaginal complications after postoperative brachytherapy in endometrial cancer: a prospective analysis

**DOI:** 10.1007/s12094-021-02737-z

**Published:** 2021-12-01

**Authors:** Y. Zhang, G. Gomez, C. Ascaso, A. Herreros, B. Fornes, J. Mases, J. Rochera, L. Tagliaferri, S. Sabater, A. Torne, A. Biete, Á. Rovirosa

**Affiliations:** 1 Cancer Center, Henan Provincial People’s Hospital, Zhengzhou, China; 2 Radiation Oncology Dpt., Hospital Los Ángeles, Chihuahua, México; 3 Fonaments Clínics Dpt., Faculty of Medicine, Universitat de Barcelona, C/Casanovas 143, 08036 Barcelona, Spain; 4 Radiation Oncology Dpt., Hospital Clinic Barcelona, Barcelona, Spain; 5 UOC Radioterapia Oncologica, Dipartimento di Diagnostica per Immagini, Radioterapia Oncologica ed Ematologia, Fondazione Policlinico Universitario Agostino Gemelli IRCCS, Rome, Italy; 6 Radiation Oncology Dpt., Hospital General Universitario de Albacete, Albacete, Spain; 7 Gynecological Cancer Unit, Hospital Clinic Barcelona, Barcelona, Spain

**Keywords:** Brachytherapy, Vaginal constraint, Vaginal complications, Postoperative endometrial cancer

## Abstract

**Purpose:**

To evaluate the preliminary results of the use of 68 Gy EQD2_(α/β=3 Gy)_ as a dose limit to the lowest dose in the most exposed 2 cm^3^ of the vagina in order to reduce G2 late vaginal problems in postoperative endometrial carcinoma (EC).

**Methods:**

From November 2016 to October 2019, 69 postoperative EC patients receiving vaginal brachytherapy (VBT) ± external beam radiotherapy (EBRT) were prospectively analyzed. The median EBRT dose was 45 Gy (range: 44–50.4 Gy), 1.8−2 Gy/day, 5 fractions(Fr)/week. VBT was administered with the following schedule: 1Fr of 7 Gy after EBRT and 2 daily Fr × 7.5 Gy in exclusive VBT. The dose was prescribed at 0.5 cm from the applicator surface with an active length of 2.5 cm; 56 patients were treated with vaginal cylinders (49–3.5 cm, 6–3 cm, and 1–2.5 cm) and 13 with the colpostat technique. The overall VBT dose was adjusted to meet the vaginal restriction of < 68 Gy EQD2_(α/β=3 Gy)_ at 2 cm^3^. Late toxicity was prospectively assessed using RTOG scores for bladder and rectum, and the objective LENT-SOMA criteria for vagina.

**Results:**

With a median follow-up of 31.0 months, no vaginal-cuff recurrences were found. Late toxicity: only 1G1(1.4%) rectal toxicity; 21G1(30.4%) and 3G2(4.3%) vaginal complications. Only one (1.4%) of 3 G2 manifested as vaginal shortening.

**Conclusions:**

In postoperative EC patients treated with VBT, only one developed G2 vaginal stenosis with the use of 68 Gy EQD2_(α/β=3 Gy)_ as a dose constraint. These preliminary results seem to indicate the value of this dose limit for reducing G2 vaginal stenosis. Nonetheless, these findings should be confirmed in a larger number of patients with longer follow-up.

## Introduction

Endometrial cancer is a common neoplasm in developed countries. In Spain, it is the fourth most frequent neoplasm in women and the first of the female genital tract, with an estimated 6784 new cases and 1660 deaths in 2018. Despite the increase in incidence, the mortality rate has decreased in Europe. Most of these neoplasms develop in postmenopausal women, with a mean age of 60 years, and the diagnosis is usually achieved in early stages considering that the most common manifestation is metrorrhagia [1, 2].

The ideal treatment for endometrial cancer is total hysterectomy with bilateral salpingo-oophorectomy. Pelvic lymphadenectomy or nodal sampling is performed in cases of high-grade tumors or on suspicion of ≥ 50% invasion to the myometrium [3–6]. Adjuvant radiotherapy is administered to patients according to the risk factors of recurrence, which are defined after pathological analysis. Adjuvant vaginal brachytherapy (VBT) is a well-established treatment, aimed at preventing tumor recurrence, with a vaginal-cuff from 15 to 2% or less according to the series. The last ESGO/ESTRO/ESP recommendations consider molecular study for treatment selection [7], and PORTEC 4a will provide more relevant information in this aspect. Since 2003 patients with intermediate-risk have been treated with exclusive VBT at Hospital Clinic of Barcelona, with 0% of vaginal-cuff recurrence and less than 2% when VBT was associated with external beam radiotherapy (EBRT). Indeed, in the last 107 patients this value was 0.9% [8, 9].

There is no established dose schedule for adjuvant treatment with VBT. The recommendations in the different series reported range from 1 to 6 fractions for exclusive brachytherapy, and 1–4 fractions after EBRT. The ABS society described 22 schedules after EBRT and 24 in exclusive treatment, and the most commonly used were 7 Gy for three fractions at a depth of 0.5 cm in monotherapy and 5 Gy for three fractions at a depth of 0.5 cm in combined treatment [10]. At Hospital Clinic of Barcelona we have reported the benefits of using 3 fractions of 6 Gy in exclusive brachytherapy and a single dose of 7 Gy after EBRT, with excellent results in local control, patient comfort and savings in costs [9, 11, 12].

Although in general, this treatment is well tolerated, few studies have analyzed vaginal toxicity, with G1-G2 being the most common, and usually only G3-4 late toxicity are mentioned to indicate the low rate of severe complications. The main factors associated with the presence of late vaginal complications, mainly stenosis, are vaginal surface doses, cylinder diameter, high dose per fraction, and active length. Other confounding factors, such as age, menopausal status, comorbidities, marital status have been described having influence in vaginal complications [13].

Three previous studies at Hospital Clinic of Barcelona reported that an EQD2_(α/β=3 Gy)_ > 68 Gy at 2 cm^3^ was associated with vaginal toxicity ≥ G2 in 20% of patients [13–15]. The last 2 preliminary studies with 2 different schedules showed an incidence of G2+ late vaginal toxicity of 24.3% and 10.7% in the schedules of 1 fraction of 7 Gy after EBRT and 3 fractions of 6 Gy for exclusive VBT, respectively, and 18.6% and 4.8% for 2 fractions of 5–6 Gy after EBRT and 4 fractions for exclusive VBT, respectively, with a follow-up of 30 and 29.5 months, respectively [8, 16]. From November 2016 to October 2019, in 69 patients we prospectively applied the restriction of the EQD2_(α/β=3 Gy)_ 68 Gy at 2 cm^3^ of the most exposed vaginal clinical target volume (CTV) in both EBRT + VBT and also in exclusive brachytherapy.

The objective of present study was to evaluate the preliminary results of the use of this constraint to the vagina to reduce G2 late vaginal problems and also the vaginal-cuff control obtained. To our knowledge, this is the first article to prospectively analyze the impact of a dose restriction for the vagina to prevent stenosis.

## Materials and methods

From November 2016 to October 2019, we prospectively recorded and posteriorly analyzed the results of vaginal late complications in 69 patients with endometrial cancer treated with VBT ± EBRT after surgery.

The preset study had institutional review board (RBI) approval (HCB/2020/0067).

After the diagnosis of endometrial cancer, imaging work-up studies, including magnetic resonance (MR), positron emission tomography, computerized tomography (CT) and/or ultrasonography were performed. The 69 patients underwent surgery with the following approaches: 35 (50.7%) underwent laparoscopic-assisted vaginal hysterectomy and bilateral salpingo-oophorectomy (LAVH-BSO) with pelvic ± para-aortic lymphadenectomy; 13 patients (18.8%) only had LAVH-BSO, and three (4.4%) had vaginal hysterectomy with bilateral salpingo-oophorectomy, and in 2 patients (2.9%) sentinel nodes were also resected. Abdominal hysterectomy plus BSO with pelvic lymphadenectomy was performed in five patients (7.2%) and para-aortic lymphadenectomy was performed in 2 patients (2.9%) and in eight patients (11.6%) omentectomy was also performed. Only one (1.4%) patient underwent simple abdominal hysterectomy.

After pathologic analysis, 35 (50.7%) patients were classified as the intermediate-risk group following the ESGO-ESTRO-ESMO classification of 2016 [6] and received exclusive VBT, while the remaining 34 (49.3%) patients underwent VBT + EBRT. Chemotherapy was administered prior to radiotherapy depending on age and comorbidities in 17 (24.6%) patients (4–6 cycles of carboplatin plus paclitaxel). Table 1 shows the pathological characteristics of the patients.

**Table 1:** Pathological characteristics of the patients included in the study (*N* = 69)

Variable			
Age in years (mean and range)		65.8	41–86
Variable		*N*	%
2009 FIGO stage			
	IA	31	44.9
	IB	15	21.7
	II	8	11.6
	IIIA	1	1.4
	IIIB	0	0
	IIIC1	6	8.7
	IIIC2	7	10.4
	IVA	1	1.4
Pathological types			
	Endometrioid	55	79.7
	Serous	4	5.8
	Clear Cells	5	7.2
	Mixed	4	5.8
	Mucinous	1	1.4
Grade			
	1	23	33.3
	2	24	34.8
	3	22	31.9
Myometrial invasion			
	≤ 50%	40	58.0
	> 50%	29	42.0
VLSI			
	No	37	53.6
	Yes	26	37.7
	NA	6	8.7

*VLSI* vascular lymphatic space invasion, *NA* not available

Thirty-four patients received EBRT, 17 of whom were treated with the intensity modulated radiation therapy and volumetric modulated arc therapy technique with 6MV and 17 patients received 18MV after 3D planning. Target volume definition was performed following the recommendations of the RTOG [17]. The median dose was 45 Gy (range: 44–50.4 Gy), and the dose per fractionation was 1.8–2 Gy/day, 5 fractions/week. All the patients received high-dose-rate (HDR)-VBT with the following schedule aim: 1 fraction of 7 Gy after EBRT and 2 daily fractions of 7.5 Gy in exclusive vaginal brachytherapy. The overall VBT dose was adjusted to meet the vaginal restriction of < 68 Gy EQD2 at 2 cm^3^ of the most exposed vaginal CTV. The brachytherapy technique, vagina CTV and contouring of organs at risk (OAR), and also 3D treatment planning have been described elsewhere [13, 14]. The treatment planning system employed was the Oncentra Brachy planning system (v. 4.1) (Elekta^®^, Nucletron BV, Veenendaal, The Netherlands). CT and MR compatible vaginal applicator set by Nucletron BV, Veenendaal (The Netherlands) was used for treatment of the vaginal cuff. This applicator is available in different cylinder diameters, but the largest comfortable for the patient was chosen to reduce the vaginal surface dose (2 cm, 2.5 cm, 3 cm, and 3.5 cm) considering each patient’s anatomy. Fifty-six patients were treated using the vaginal cylinder technique (diameter of 3.5 cm in 49 patients, 3 cm in 6 patients, 2.5 cm. in 1 patient), and the 13 remaining patients were treated with the colpostat technique (diameter of 2 cm in 2 patients, 2.5 cm in 6 patients and 3 cm in 5 patients). The colpostat technique was chosen for patients with small introitus and a wide vaginal-cuff vagina. Table 2 shows the characteristics of EBRT and brachytherapy.

**Table 2:** Brachytherapy dose characteristics (*N* = 69)

Treatment modality	Dose per fraction(Gy)	Number of patients
Exclusive VBT	7.25	1 (1.4%)
	7.5	34 (49.3%)
EBRT + VBT	5.75	1 (1.4%)
	6	1 (1.4%)
	6.2	1 (1.4%)
	6.25	3 (4.3%)
	6.3	1 (1.4%)
	6.4	1 (1.4%)
	6.5	18 (26.1%)
	6.7	1 (1.4%)
	6.75	4 (5.8%)
	7	3 (4.3%)

*VBT* vaginal brachytherapy, *EBRT* external beam radiation therapy

The vaginal brachytherapy dose was prescribed at 0.5 cm from the applicator surface with optimization to points and with an active source length of 2.5 cm. 90% isodose was considered to cover all the CTV of the vagina whenever possible. Following treatment, the patients were strongly encouraged to use the vaginal dilators. Follow-up was carried out by the same radiation oncologist every 3–4 months with clinical and also imaging studies when necessary during the first 2 years and every 6 months thereafter. Late toxicity was prospectively assessed using RTOG scores for the bladder and rectum, and the objective criteria of LENT-SOMA for vagina (Table 3) [18, 19].

**Table 3:** Late toxicity by the objective late effects of normal tissues—subjective, objective, management, analytical (LENT-SOMA) criteria for the vagina

G1	Atrophy, telangiectasia adherences, < 1/3 shortened vaginal length
G2	Bleeding telangiectasias, symptomatic dryness, 1/3 and 2/3 shortened vaginal length, partial synechiae
G3	Vaginal length < 1/3, deep ulceration, complete synechiae
G4	Obliteration, fistula, persistent bleeding

EQD2_(α/β=3 Gy)_ at the vaginal surface was calculated using a point located at the applicator surface at the level of the prescription point, being the sum of the VBT ± EBRT dose. EQD2_(α/β=3 Gy)_ at 2 cm^3^ of the most exposed part of vaginal CTV was calculated, and the position was always in the most cranial part of the applicator. In patients receiving combined treatment, EQD2 _(α/β=3 Gy)_ of EBRT was added to the EQD2 _(α/β=3 Gy)_ VBT dose. The procedure of CTV definition and dose prescription has previously been reported [15].

Statistics: All the data were analyzed descriptively by frequency, maximum, minimum, average, median and standard deviation tables.

## Results

The median and mean follow-up of the patients were 31.0 months and 33.5 months (13–62, SD 10.6), and the mean age of diagnosis was 65.6 years.

No vaginal-cuff recurrence was found in the 69 patients, but 11 developed regional and distant recurrences. At the time of the analysis, there were 9 deaths, 7 related to endometrial cancer, 1 due to previously known renal failure, and the remaining death was due to pancreatic cancer.

Table 4 shows the D90 of the CTV and EQD2_(α/β=3 Gy)_ at 2 cm^3^ of the most exposed volume of CTV.

**Table 4:** D90 and D2 cm^3^ EQD2_(α/β = 3 Gy)_ at CTV

	D90 per VBT fraction (Gy)		D2 cm^3^ EQD2_(α/β=3 Gy)_(Gy)	
	Exclusive BT	EBRT + BT	Exclusive BT	EBRT + BT
Mean	8.57	7.24	60.6	66.6
Median	8.62	7.49	60.1	67.1
Minimum	6.75	5.72	54.5	61.2
Maximum	9.49	10.59	66.4	67.8
SD	0.59	0.91	3.35	1.41

*SD* standard deviation; *BT* brachytherapy; *EBRT* external beam radiation therapy

No patient presented late bladder toxicity at the time of the present analysis, and only 1 patient developed late G1 rectal toxicity. In relation to late vaginal toxicity, 45 (65.2%) patients did not develop any vaginal toxicity, while 21 patients (30.4%) and 3 patients (4.3%) developed G1 and G2 complications respectively. Of these patients with G1 toxicity, telangiectasia was manifested in 12 patients (17.4%), < 1/3 shortened vaginal length was manifested as a “dog ear” in 5 patients, and vaginal adhesions were observed in 4 patients (5.8%). Two patients presented G2 toxicity in the form of bleeding on contact of telangiectasias at examination, and the remaining patient presented > 1/3 shortened vaginal length.

The mean and median EQD2_(α/β=3 Gy)_ to the most exposed 2 cm^3^ of vagina in G0 patients was 63.9 Gy and 65.9 Gy (54.5–67.8, SD 3.92), 63.1 Gy and 64.1 Gy (55.4–67.7, SD 4.23) in G1, 61.3 Gy and 61.2 Gy (60.8–61.8, SD 0.50) in patients with G2 toxicity. Two of 3 G2 patients were from the exclusive brachytherapy group treated with a 3 cm diameter cylinder, and the remaining patient with vaginal shortening was from the combined treatment group treated with a 3.5 cm diameter cylinder.

Patient compliance with vaginal dilator use was not very high. Twenty-nine patients (42%) used the vaginal dilators for 2 years or more, 34 patients (49.3%) failed to comply less than 9 months and no information was available for the remaining 6 patients (8.7%). Patients with G2 problems did not use the vaginal dilators, or they were used for less than 9 months.

## Discussion

Since the introduction of HDR BT in the 1960s, this alternative treatment for endometrial cancer has been popularly adopted by many radiation oncologists. The incorporation of three-dimensional (3D) treatment planning makes gross tumor volume (GTV) and CTV coverage more accurate and a better normal tissue sparing is obtained. Some analyses reported an equal efficacy and safety profile in patients receiving either HDR or low dose rate (LDR) BT [20–22]. In contrast to the LDR technique, HDR application is preferable because of shorter administration time, no need for hospitalization, reduced morbidity, lower cost, possibility of dose optimization, and patient comfort.

Owing to the low incidence of severe toxicity by postoperative BT in endometrial cancer, few studies have focused on this topic. However, as a frequent vaginal side effect, vaginal stenosis/shortening can lead to sexual dysfunction, psychosocial problems, and can even impede gynecologic examination. Vaginal dose is an acknowledged risk factor for long-term vaginal toxicity, but no dose constraint has been established in 3D BT.

GEC-ESTRO and other groups recommend EQD2_(α/β=3 Gy)_ at the most exposed 2 cm^3^ of normal tissue as a limit for bladder, rectum and sigmoid [23, 24]. Hence, we hypothesized that considering the vagina as an OAR, EQD 2 cm^3^ values of vagina could be designated as a predictor of vaginal toxicity. On analyzing the correlation of dose with toxicity in postoperative patients treated with HDR BT, we found that there was an association between EQD2_(α/β=3 Gy)_ at 2 cm^3^ of vagina and G2 late vaginal toxicity [13–15]. A dose limit of 68 Gy EQD2_(α/β=3 Gy)_ at the most exposed 2 cm^3^ of the vagina has been applied for reducing vaginal toxicity since 2016. In the present study, 69 consecutive patients were included to evaluate the effect of BT with this constraint. The results showed that with the restriction of vaginal dose, the incidence of G2 vaginal toxicity was 4.3%, and only one (1.4%) patient with G2 vaginal toxicity presented vaginal shortening; thereby, demonstrating its value in preventing G2 vaginal shortening.

In the previous studies by our group, the incidence of G2 vaginal toxicity varied from 10.4 to 21.5%, with an incidence of vaginal shortening of 9.7% [9, 13, 14]. This shows that G2 vaginal toxicity was reduced by limiting the EQD2_(α/β=3 Gy)_ at 2 cm^3^ of the vagina. With the new schedule of 2 fractions of 7.5 Gy in exclusive brachytherapy, the EQD2_(α/β=10 Gy)_ at the vaginal surface with a cylinder diameter of 3.5 cm is 41.2 Gy, while with the 3 fractions of 6 Gy previously used in our center the dose was 44.1 Gy. Regardless of the different schedules used, excellent local control was achieved. One study by Damast showed the same result with 2 fractions of 7 Gy [25]. In addition, one fraction of 7 Gy after EBRT has shown to be as effective in local control as other regimens [9]. Similarly, a 5 year local control rate of 93% was reported with the schedule of a dose of 7 Gy for VBT boost in HDR prescribed at 5 mm from the applicator surface with the proximal half to two-thirds of the vagina being irradiated [20].

In the present study, only one patient receiving exclusive BT needed one dose per fraction reduction, but after EBRT only 3 patients received 7 Gy, and the rest required a dose reduction, the most common being a dose ≤ 6.5 Gy. These are preliminary results, but in 2 years we will likely have more robust results. In any case, these results suggest that a dose reduction per fraction is possible in order to reduce complications in postoperative brachytherapy. Another aspect to be determined in these treatments after establishing a homogeneous CTV definition is the determination of the best D90 as in other brachytherapy treatments.

Vaginal stenosis has a negative impact on patient quality of life. With regard to sexual dysfunction, Akbaba reported that sexual activity and enjoyment decreased after irradiation and the higher the degree of vaginal stenosis, the less the sexual enjoyment [26]. Similarly, in a study by Schover, greater vaginal narrowing and shortening tended to produce greater dyspareunia and postcoital soreness. Moreover, psychological status, daily life activities and pelvic examination are also negatively affected [27].

For the prevention of vaginal stenosis, guidelines and studies recommend the use of a vaginal dilator, but this is not supported by good quality evidence. The same study by Akbaba showed that vaginal dilator use did not reduce the incidence of vaginal stenosis [26]. A Cochrane systematic review, reported the absence of reliable evidence to demonstrate that vaginal dilation prevents stenosis [28]. However, another study found that dilator use at least two to three times a week was associated with a decreased risk of vaginal stenosis [29]. Stahl et al. reviewed 243 endometrial cancer patients treated with brachytherapy to evaluate the effect of vaginal dilator use, and reported that extended compliance was a significant predictor of reduced risk of vaginal stenosis [30]. In addition, low vaginal dilator compliance makes it difficult to guarantee that vaginal stenosis can be prevented. Although in the present study patient compliance with vaginal dilator use was not very high, dilators, and intercourse are highly recommended to minimize the risk of vaginal stenosis. This confounding finding could have a significant effect on the results. In the present study, we found that all G2 patients either did not use the dilator or used it for less than 9 months.

Our present study found excellent results in terms of vaginal stenosis with the application of the present dose constraint, although the limitations include the small sample size and the preliminary nature of the results. We expect to obtain more robust conclusions with an increase in the sample size and a longer follow-up. The next step in the future is to perform a prospective multicenter trial analyzing the effect of administering < 68 Gy EQD2 _(α/β=3)_ to the most exposed 2 cm^3^ of vaginal CTV as a dose constraint.

## Conclusions

Following the preliminary results of postoperative endometrial cancer treated with brachytherapy with 68 Gy EQD2_(α/β=3 Gy)_ at the most exposed 2 cm^3^ of vagina as a dose constraint, only one patient developed G2 vaginal stenosis; thereby, indicating the possible value of this dose limit for reducing G2 vaginal stenosis.
